# Gold nanoparticle-based rapid detection and isolation of cells using ligand-receptor chemistry

**DOI:** 10.1038/s41598-018-21068-8

**Published:** 2018-02-13

**Authors:** Pradipta Ranjan Rauta, Pavan M. Hallur, Aditya Chaubey

**Affiliations:** Anti-Cancer Technologies Program, Mazumdar Shaw Center for Translational Research, NH Health City, Bangalore, 560 099 India

## Abstract

Identification and isolation of low-frequency cells of interest from a heterogeneous cell mixture is an important aspect of many diagnostic applications (including enumeration of circulating tumor cells) and is integral to various assays in (cancer) biology. Current techniques typically require expensive instrumentation and are not amenable to high throughput. Here, we demonstrate a simple and effective platform for cell detection and isolation using gold nanoparticles (Au NPs) conjugated with hyaluronic acid (HA) i.e. Au-PEG-HA NPs. The proposed platform exploits ligand-receptor chemistry to detect/isolate cells with high specificity and efficiency. When the Au-PEG-HA NPs come in contact with cells that express CD44 (the receptor for HA), a clear colorimetric change occurs (along with an accompanying SPR peak shift from 521 nm to 559 nm) in the solution due to NPs-cell interaction. This clearly discernible, colorimetric change can be leveraged by point-of-care devices employed in diagnostic applications. Finally, we show that we can successfully isolate viable cells from a heterogeneous cell population (including from human blood samples) with high specificity, which can be used in further downstream applications. The developed NPs-based platform can be a convenient and cost-efficient alternative for diagnostic applications and for cell isolation or sorting in research laboratories.

## Introduction

Isolation and characterization of rare or low-frequency cells of interest from a heterogeneous population is of critical significance in many biomedical applications. Typically, this is accomplished via techniques like differential centrifugation, or through instrumentation such as Fluorescence-Activated Cell Sorting (FACS) and Magnetic Activated Cell Sorting (MACS). However, these techniques are not amenable to high throughput and resolution and are also time-consuming. Crucially, both FACS and MACS require sophisticated instrumentation, a high level of technical expertise and are also prohibitively costly^1–3^. These issues are especially relevant in resource-constrained labs in developing countries^4,5^.

The key challenge that persists with conventional techniques is the process of tagging the labelling molecule, i.e., proper binding of any foreign ligand to the receptor of interest so as to increase detection sensitivity. In order to develop an easy and reliable method of cell isolation, issues such as the viability of recovered cells and cell purity need to be addressed^2,6,7^.

Nanoparticle-based platforms are amenable to easy labelling and rapid cell capture, isolation of low-frequency cells, efficient cellular manipulation, sorting, and enumeration based on their unique structural and functional properties that are not present in larger molecules^2,3,8,9^. Therefore, nanoparticle-based platforms offer a new avenue for rapid, low-cost and extremely sensitive detection of specific cells in a heterogeneous population.

Colorimetric nano-biosensors with engineered nanoparticles have the potential to detect specific cell types for different disease diagnosis^10,11^. Gold nanoparticles (Au NPs) are used widely in various biological applications due to their unique optical properties. Au NPs are easy and economical to work with due to their relatively easy synthesis, facile surface chemistry, excellent biocompatibility, spectral properties and a prominent surface plasmon resonance (SPR) peak that gives rise to a sharp and intense absorption band in the visible range^12^. Efficient target interaction can be achieved due to the large surface-to-volume ratio of Au NPs, which can further be exploited to develop new assays with ultra-sensitivity and multiparametric capabilities^13^. Typically, Au NP applications are mainly based on the level of aggregation due to NPs-target moiety interaction, which in turn leads to a significant change in the spectral properties (color change observed in the NPs solution)^14^. This colorimetric information circumvents the relative complexity that is intrinsic to optical imaging/detection approaches.

Functionalization of NPs is a widely used technique that allows its conjugation with ligands, leading to selective binding to specific cell types. The conjugation of Au NPs to monoclonal antibodies with high affinity makes them useful as biosensors^15,16^. However, antibody orientation on the surface of the NPs is crucial for effective diagnostic response^17,18^. This is an issue because of the presence of multiple reactive functional groups on antibodies, which may lead to heterogeneous antibody orientations on the NPs, resulting in nonspecific interaction^16,19^. Additionally, the conformational stability of an antibody is low and they are also prone to degradation, which can limit their utility in non-laboratory diagnostic environments^20^. Further, the relatively high cost of antibodies makes working with them an expensive proposition. Therefore, alternative ligands such as small molecules are getting increasing attention due to their stability, ease of conjugation with NPs and cost effectiveness^16^.

The cell surface glycoprotein CD44 is a promising target molecule as a diagnostic marker for cancer^21^ and as a target for therapeutic intervention^22,23^. Due to the strong binding of CD44 with its ligand, hyaluronic acid (HA), it stands to reason that CD44-HA interaction can serve as a potential diagnostic tool to efficiently aid early diagnosis of cancer^21,24^. HA, a small molecule^25^, is a water-soluble, non-immunogenic polysaccharide, making it a potential candidate for use as a ligand for CD44 for various applications.

Here, we describe the fabrication of a simple and effective platform for cell detection and isolation using Au NPs conjugated with hyaluronic acid (HA). These NPs selectively bind to the cells expressing the CD44 receptor, demonstrating CD44-HA receptor-ligand specificity. The NPs upon binding to the CD44-expressing cells aggregate and exhibit color change and show a distinct SPR peak shift. These NPs can be used to effectively separate the cells of interest from a heterogeneous cell population by differential centrifugation. The resulting pellet allows for a high percentage recovery of cells of interest, demonstrating the high specificity and robustness of the developed NPs platform. The recovered cells are viable and can be cultured for further downstream experiments.

## Materials and Methods

### Materials

Hydrogen tetrachloroaurate (III) (≥99.9%) (Catalogue no. 50790), sodium citrate tribasic dehydrate (≥99.0%) (Catalogue no. 54641), hyaluronic acid (Molecular weight- 1.5 to 1.8 × 10^6^ Da; catalogue no. 53747), polyethylene glycol (average Mn 400; catalogue no. 202398), 1-ethyl-3-(-3-dimethylaminopropyl) carbodiimide hydrochloride (EDC) (catalogue no. E7750), and N-hydroxysuccinimide (NHS) (catalogue no. 130672) were purchased from Sigma-Aldrich and used without further purification. Dulbecco’s Modified Eagle’s medium (DMEM; catalogue no.11995065), Fetal Bovine Serum (South American origin FBS; catalogue no.10270106), Penicillin-Streptomycin (catalogue no. 15140122), Phosphate buffered saline (PBS; catalogue no: 10010023) and Trypsin-EDTA (catalogue no. 25200056) were procured from GIBCO, Invitrogen, USA. ACK lysing buffer (catalogue no. A1049201) was purchased from Thermo Fisher Scientific, USA.

### Synthesis and functionalization of Au NPs

Citrate-capped Au NPs were prepared according to a modified Frens method as described previously^26,27^. Briefly, an aqueous solution of chloroauric acid (HAuCl_4_; 0.25 mM, 50 ml) was heated under reflux and 1.3 ml solution of sodium citrate (1%) with or without PEG 400 (25 µg/ml) was added to it under vigorous stirring. The solution was boiled for 15 minutes, resulting in PEGylated Au NPs. The PEGylated Au NPs were then conjugated to HA via EDC/NHS coupling. EDC [1-ethyl-3-(-3-dimethylaminopropyl) carbodiimide hydrochloride] is a common carbodiimide which catalyzes the formation of amide bonds between carboxy and amine groups. NHS (N-Hydroxy succinimide) is used to increase the stability of active intermediates in coupling reactions. This coupling via the EDC/NHS chemistry provides a covalent bond without the addition of a spacer^28^. Briefly, 50 mg of desalted HA was dissolved in 5 ml of NaHCO_3_/acetic acid buffer solution (pH 4.7), stirred for 2 hours (400 rpm) and then activated with a mixture of NHS and EDC (molar ratio of NHS/EDC is 0.25). The reaction was allowed to continue for 3 hours with constant stirring (600 rpm) at room temperature. Subsequently, Au-PEG NPs were added into reaction mixture to obtain HA/NH_2_-PEG molar ratios of 1.0. The reaction was performed for 48 hours under constant stirring (600 rpm) at room temperature. The resulting product (Au-PEG-HA NPs) was purified by dialysis (12 kDa MWCO) against distilled water for 24 hours.

### Characterization studies

Conjugation of PEG and HA with Au NPs was confirmed by FTIR spectroscopy and NMR spectroscopy, respectively. ATR-FTIR was performed on an Agilent CARY 630 FTIR spectrometer (Agilent Technologies, USA) with a resolution of 4 cm^−1^. The samples (25 µl) were scanned in the spectral region between 4000 and 400 cm^−1^ by taking an average of 25 scans. The results thus obtained were analyzed through Origin8 software. For NMR spectroscopy, 5–10 mg of Au-PEG-HA NPs and HA were dissolved in 1 ml of deuterium oxide. 1H-NMR spectra were collected at 35 °C in deuterium oxide at a frequency of 500 MHz using an NMR spectrometer (Bruker AV III) with a single axis gradient inverse probe. The colloidal Au NPs, Au-PEG NPs, Au-PEG-HA NPs were also characterized by using ultraviolet-visible spectroscopy (UV-Vis), transmission electron microscopy (TEM), dynamic light scattering (DLS) measurements. The UV-Vis spectra of the synthesized Au-PEG NPs and Au-PEG-HA NPs were recorded on a UV-Vis spectrometer (Multiskan™ GO Microplate Spectrophotometer, Thermo Fisher Scientific), using standard quartz cells at room temperature, over a spectral range between 350 nm to 900 nm at a spectral resolution of 2 nm. For TEM examination, 5 *μ*L of aqueous suspensions (10^2^ dilutions) containing Au NPs, Au-PEG NPs and Au-PEG-HA NPs were deposited on carbon-coated copper grids. After 15 minutes, excess water was removed by filter paper and the samples were left to dry at room temperature. TEM images were taken on a Jeol JEM 1010 transmission electron microscope (JEOL Ltd., Tokyo, Japan), equipped with a Mega VIEW III camera (Olympus, Soft Imaging System, Germany), operating at 80 kV. TEM results were analyzed by Image J software to evaluate the size distribution of the NPs. The DLS measurements were carried out on a Zetasizer Nano-ZS (Malvern Instruments, Worcestershire, UK).

### Cell culture

The cell lines chosen for NPs–cell interaction were MDA-MB-231 (CD44^+ve/high^), BT-474 (CD44^−ve/low^) and NIH 3T3 (CD44^−ve^). The MDA-MB-231-GFP cell line used for FACS experiments was procured from Cell Biolabs, Inc., CA, USA (catalogue no. GFPAKR210). MDA-MB-231 and BT-474 cancer cell lines were generous gifts from Prof. Annapoorni Rangarajan, Indian Institute of Science, Bangalore, India. NIH 3T3 cell line was a kind of gift from Dr Arka Shubra Ghosh, GROW labs, Narayana Netralaya, Narayana Health City, Bangalore, India. The basal medium used for all cell culture experiments was Dulbecco’s Modified Eagle’s medium supplemented with 10% Fetal Bovine Serum, and antibiotics streptomycin sulfate and benzyl at final concentrations of 100 µg/ml and 100 U/ml respectively. Cell cultures were maintained at 37 °C at 5% CO_2_ throughout all experiments. The cells were passaged using 0.25% Trypsin-EDTA at around 80% confluency. All the cells used in this study were tested for mycoplasma contamination by 4, 6-diamidino-2-phenylindole (DAPI) staining, and were found to be negative.

### Standardization of gold and hyaluronic acid concentration

Au-PEG NPs were serially diluted in a 96 well ELISA plate (concentration Au: 100–3.125 nM). 50,000 cells (either MDA-MB-231 or BT-474) were added to each well and incubated for 10 minutes at room temperature. Optimal Au concentration was evaluated based on the change in the color of the Au-PEG-HA NPs solution and the associated shift in the absorbance spectrum peak in the UV-Vis spectrum.

Similarly, standardization of the optimal concentration of hyaluronic acid (HA) was carried out by varying the HA concentration (HA: 400–1.56 µg/ml) while keeping the Au concentration optimized at 25 nM. 50,000 cells (either MDA-MB-231 or BT-474) were added to each well and incubated for 10 minutes at room temperature. Optimal HA concentration was evaluated based on the change in the color of the Au-PEG-HA NPs solution, and the associated shift in the absorbance spectrum peak in the UV-Vis spectrum.

### UV-Visible spectrum analysis of Au-PEG-HA NPs upon addition of cells

The interaction of Au-PEG-HA NPs with different cells (at varying numbers) was studied as a function of colorimetric changes as well SPR peak shift. The cells (MDA-MB-231, BT-474, NIH 3T3) were harvested and suspended in PBS. The cells were then serially diluted in 200 µl PBS ranging from 50,000 to 195 cells. 100 µl of Au-PEG-HA NPs (25 nM Au and 100 µg/ml HA) solution was added to each well and allowed to incubate for 10 minutes at room temperature. The observed colorimetric change and UV-Vis absorption spectra were recorded and compared with the Au-PEG-HA NPs without cells.

The influence of **c**ell number on the aggregation of the Au-PEG-HA NPs was investigated by monitoring the changes in the absorbance ratio at 650 and 521 nm (A650/A521 ratio) to develop a quantitative estimation of the number of cells bound to NPs.

All the experiments were performed in triplicates. For each set of experiments, the NPs were freshly synthesized and the data is provided with the standard deviation.

### Processing of blood sample

Blood samples were obtained from Mazumdar Shaw Medical Center, Bangalore (Narayana Health Medical Ethics Committee approval no. NHH/MEC-CL-2017-457). All the experiments were performed in accordance with the guidelines and regulations mentioned in the Ethics Committee approval and all the samples were collected with informed consent of the participants. 10 ml of blood sample was collected in 10% (w/v) sodium citrate by venipuncture from each healthy volunteer. 1 ml of whole blood was mixed with 10 ml of ACK lysis buffer for 5 minutes at room temperature to lyse red blood cells (RBC). After RBC lysis, the white blood cells were collected by centrifuging the mix at 1800 rpm for 5 minutes. The supernatant was aspirated and white blood cells were suspended in PBS.

### Evaluation of the specificity of Au-PEG-HA NPs interaction with cells of interest in a heterogeneous population

To evaluate the efficacy of functionalized Au-PEG-HA NPs in selectively binding to cells expressing CD44, cell suspensions were prepared by mixing MDA-MB-231 (CD44^+ve/high^) and BT-474 cells (CD44^−ve/low^) in varying ratios, but keeping the total number of cells constant (2000) in each well in 200 µl of PBS. Next, 100 µl of Au-PEG-HA NPs solution (25 nM Au and 100 µg/ml HA) was added to each well followed by incubation for 10 minutes at room temperature. The colorimetric changes and UV-Vis absorption spectra were recorded and compared with control (Au-PEG-HA NPs) values using the A650/A521 ratio.

In another experiment, individual cell types i.e. MDA-MB-231/BT-474/NIH3T3/Blood (5000 cells in 200 µl PBS) were mixed with Au-PEG-HA NPs (100 µl) and allowed to incubate for 10 minutes at room temperature. The cells bound to Au-PEG-HA NPs were then separated by density gradient method using differential centrifugation at 600 rpm for 30 min. The cell pellet and the supernatant were later separated and subjected to flow cytometry for cell-count analysis. Flowing Software was used for determining the percentage of cells captured in both the supernatant and the pellet. The analysis was performed to calculate the percentage of cells in the pellet and supernatant for MDA-MB-231/BT-474/NIH3T3/Blood.

All the experiments were performed in triplicates. For each set of experiments, the NPs were freshly synthesized and the data is provided with the standard deviation.

### Isolation and recovery of cells from a heterogeneous population

To evaluate the cell recovery efficiency and viability, MDA-MB-231 cells were mixed with BT-474/NIH 3T3/Blood at varying densities (50%, 5% and 1% of MDA-MB-231 cells). 100 µl of Au-PEG-HA NPs solution was added to the cell suspension, followed by a 10-minute incubation at room temperature. The cells attached to the Au-PEG-HA NPs were isolated by centrifuging at 600 rpm for 30 minutes^29^. The viability (live/dead) of recovered cells in the pellet was evaluated by the trypan blue assay.

To further validate the isolation and recovery of cells of interest from a heterogeneous population, a separate experiment was designed to determine the specific cell capture efficiency of Au-PEG-HA NPs, where GFP-expressing MDA-MB-231 cells were spiked into BT-474/NIH3T3/Blood cell suspension at varying spiking densities (50%, 5% and 1% of MDA-MB-231 cells), the cells bound to Au-PEG-HA NPs were isolated using differential centrifugation. The cell pellet and the supernatant were separated and analyzed by flow cytometry using FITC filter by BD Canto II. Flowing Software was used to determine the percentage of MDA-MB-231-GFP cells captured in both the supernatant and the pellet. The analysis was performed to calculate the percentage of cells both in the pellet and in the supernatant for MB-231-GFP, BT-474, NIH3T3 and Blood cells respectively.

To further validate the viability of isolated cells, the cells from the pellet were suspended in culture medium and seeded on culture plates. After 24 hours of incubation, the cultures were evaluated for GFP-expressing MDA-MB-231 cells using Zeiss Axio Vert A1 inverted fluorescence microscope. 8–10 images were captured randomly from culture plates using both the GFP filter and brightfield microscopy. ImageJ 1.51j8 software was used to overlay images captured in the bright-field and GFP filter to visualize the morphology of the cells after 24 hours.

All the experiments were performed in triplicates. For each set of experiments, the NPs were freshly synthesized and the data is provided with the standard deviation.

## Results and Discussion

Isolation and characterization of rare cells and molecules from a heterogeneous population is of critical importance in many research and diagnostic applications. Current protocols for cell isolation techniques are based on two principles: (a) physical properties, with methods including density gradient centrifugation, membrane filtration and microchip-based capture platforms; and, (b) cellular/biological characteristics, comprising affinity methods, such as affinity solid matrix (beads, plates, fibers), fluorescence-activated cell sorting (FACS), and magnetic-activated cell sorting (MACS)^2,30^. FACS is one of the most established approach for isolation of cells from a heterogeneous population^31^. But, it requires labelling of cell surface receptor as well as a huge starting number of cells (more than 10,000) in suspension^2^. Also, the rapid flow-rate and nonspecific fluorescent molecules (e.g. auto fluorescence) can damage the viability of the sorted cells, rendering the isolation a failure^2,32^. Magnetic -based cell sorting is another common and useful approach which utilizes antibody specificity to separate cells of interest from heterogeneous cell populations^33,34^. But, this magnetic interaction is known to have a negative impact on cell viability, phenotypic identity, cell function and can present nonspecific cell-particle adhesion^32,35–37^. Adhesion-based cell isolation strategies (immobilization with capture agents, including antibody and nucleic acid aptamers) especially those combined with several microfluidic separation technologies have demonstrated the ability to isolate rare cells for clinical applications^38–41^. However, approaches like FACS, MACS, and microfluidics involve sophisticated microfabrication, expensive lab equipment, long processing time and require expertise.

The current study was designed for showcasing, as a “proof-of-concept”, the applicability of employing Au-PEG-HA NPs as a platform for isolating cells of interest from a heterogeneous population. In the present research, we have shown that our platform can be used easily and reliably to detect and isolate cells at low cost in resource-constrained conditions, with high efficiency, specificity and cell recovery by exploiting ligand-receptor binding chemistry using Au NPs. This NPs-based platform can also be very useful for single cell isolation even at low starting cell numbers (below 10,000), unlike FACS. This platform could be a viable alternative to antibody-mediated targeting of cells by minimizing issues like high cost and the possibility of nonspecific chemical interactions.

### Synthesis and characterization of the Au-PEG-HA NPs

**Figure 1:**
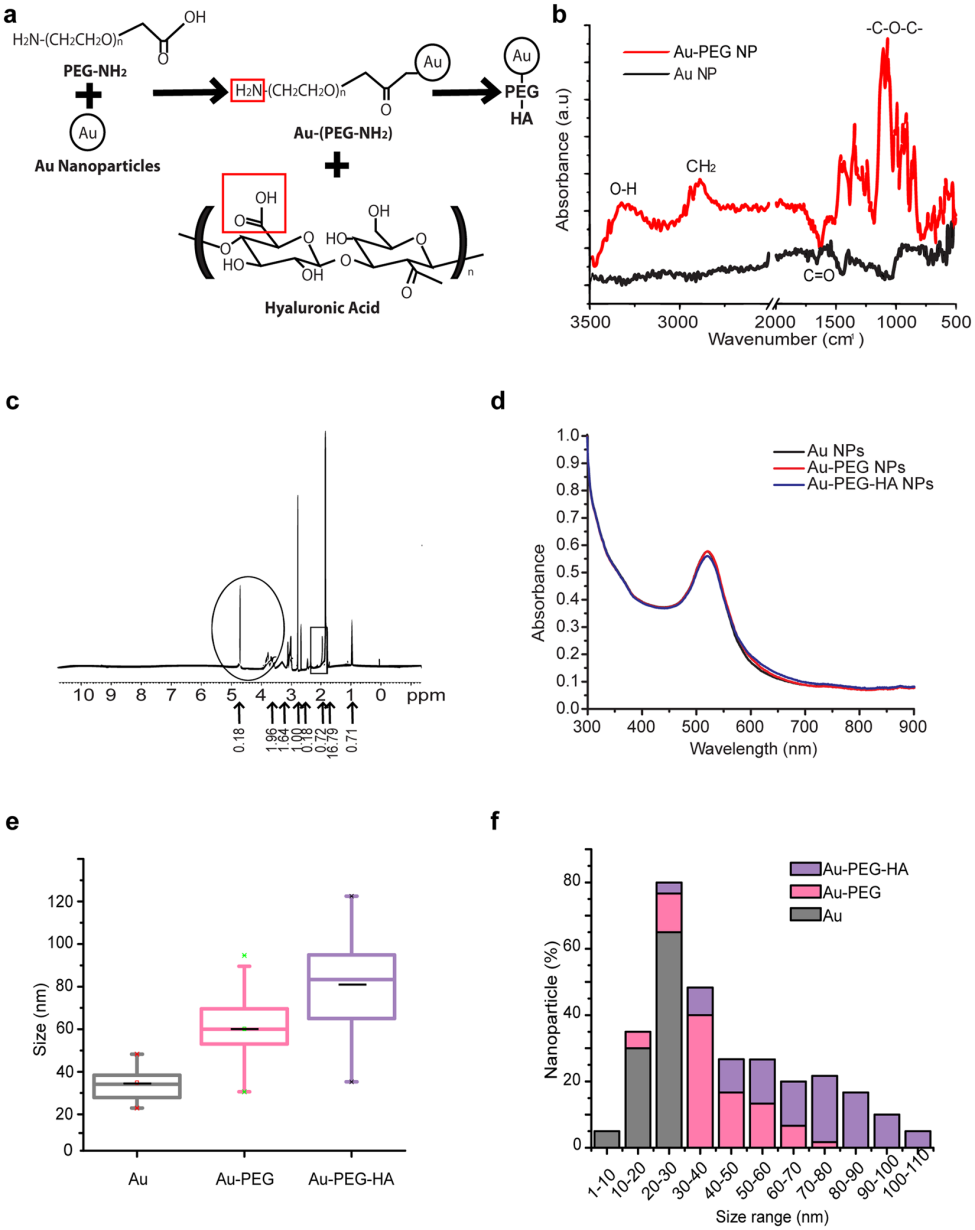
Physico-chemical properties of Au NPs, Au-PEG NPs, Au-PEG-HA NPs. (**a**) Schematic representation of the synthesis of Au-PEG-HA NPs. (**b**) ATR-FTIR spectrum of Au NPs and Au-PEG NPs depicting PEGylation of Au NPs (3347.17 cm^−1^- OH group, 1095 cm^−1^- C-O-C, 1637 cm^−1^- C = O stretching). (**c**) 1H NMR spectroscopy demonstrating the chemical structure of HA in Au-PEG-HA NPs with characteristics peak labeled (inset mentioned in Fig. S1a). (**d**) UV absorption spectra with a characteristic SPR peak (519–521 nm) of Au NPs, Au-PEG NPs, Au-PEG-HA NPs. (**e,f**) Average size analysis of Au NPs, Au-PEG NPs, Au-PEG-HA NPs determined by TEM image analysis.

Au-PEG-HA NPs were synthesized by conjugating Hyaluronan to PEGylated Au NPs as per the schematic showed in the Fig. 1a. Citrate-capped Au NPs were prepared according to a modified Frens method [See Methods]. The Au NPs were functionalized with PEG (PEGylation) to increase the hydrophilicity as well as the stability of the Au NPs (by preventing aggregation), and to provide active functional groups (-NH_2_) required for HA conjugation. The Au-PEG NPs, in contrast to Au NPs, are stable at room temperature without any change in their properties, indicating that PEG is an efficient stabilizer for Au NPs (Fig. S1). FTIR analysis confirmed PEG capping on gold NPs (Au-PEG NPs) by showing the signature peaks of PEG at 3444.17 cm^−1^ (O-H), 1095 cm^−1^ (C-O-C stretching) and 1637 cm^−1^ (C = O stretching) (Fig. 1b). Subsequent surface modification of Au-PEG NPs with HA was confirmed by 1H-NMR spectra demonstrating its chemical structure with characteristic peaks labeled i.e. broad signal (3.0–3.8 ppm): protons in the sugar rings; 4.6 ppm: two anomeric protons attached to the carbons adjacent to the two oxygen atoms; 2.02 ppm: methyl (-CH_3_) protons of the N-acetyl group of HA (Fig. 1c and inset in Fig. S2a). In homogenous samples, all the three groups of NPs exhibit the same optical response upon exposure to light. All the three NP groups were pinkish in color with a characteristic surface plasmon resonance (SPR) peak located at 519–521 nm in UV-Vis spectra. Upon conjugation of HA to Au-PEG NPs, the SPR peak showed a slight shift from 519 to 521 nm and there was no change in color of the solution, indicating unaltered optical properties of Au NPs (Fig. 1d). The size of Au NPs, Au-PEG NPs and Au-PEG-HA NPs was characterized by DLS and TEM studies (Fig. 1e and Fig. S2b,c). The average size of Au NPs, Au-PEG NPs, Au-PEG-HA NPs, assessed by TEM, was found to be 21.95 ± 6.4 nm, 47.21 ± 15.54 nm and 67.98 ± 20.4 nm respectively (Fig. 1e). The increase in the size of Au NPs upon PEGylation, followed by HA conjugation was observed and further supported via DLS analysis. The hydrodynamic diameter of Au NPs, Au-PEG NPs and Au-PEG-HA NPs was 16.53 nm, 37.18 nm and 67.95 nm as evaluated via DLS analysis (Fig. S2c). The range of size distribution of Au NPs, Au-PEG NPs and Au-PEG-HA NPs was 10–35 nm, 18–82 nm and 22–110 nm respectively (Fig. 1f). So, considering the lower margin, it can be inferred that ~18% particles are present in 1–30 nm range in case of Au-PEG NPs. Similarly, in case of Au-PEG-HA NPs, ~3% particles are present in the range 1–30 nm and ~18% particles are present in the range of 30–50 nm and ~79% of the particles were present in the 50–110 nm range (Fig. 1f). The varied size of particles present in the Au-PEG-HA NPs alludes to the inefficiency of the conjugation process. Further optimization of this process might lead to a narrower particle size distribution due to uniform distribution of HA on the surface of PEGylated Au NPs.

### Optimization of Au and HA concentration in Au-PEG-HA NPs for cell detection

We hypothesized that when the NPs come in contact with cells that express the receptor for HA (CD44), it will lead to a color change (and an accompanying absorbance shift) in the solution due to NPs-cell interaction, leading to an aggregation of Au-PEG-HA NPs.

Therefore, our next step was to optimize the concentration of both Au and HA in Au-PEG-HA NPs group to allow for clear visualization of the colorimetric changes induced by its binding to the cells of interest. For this, we utilized two cell lines that express varying levels of CD44: MDA-MB-231 (CD44^+ve/high^) and BT-474 (CD44^−ve/low^).

**Figure 2:**
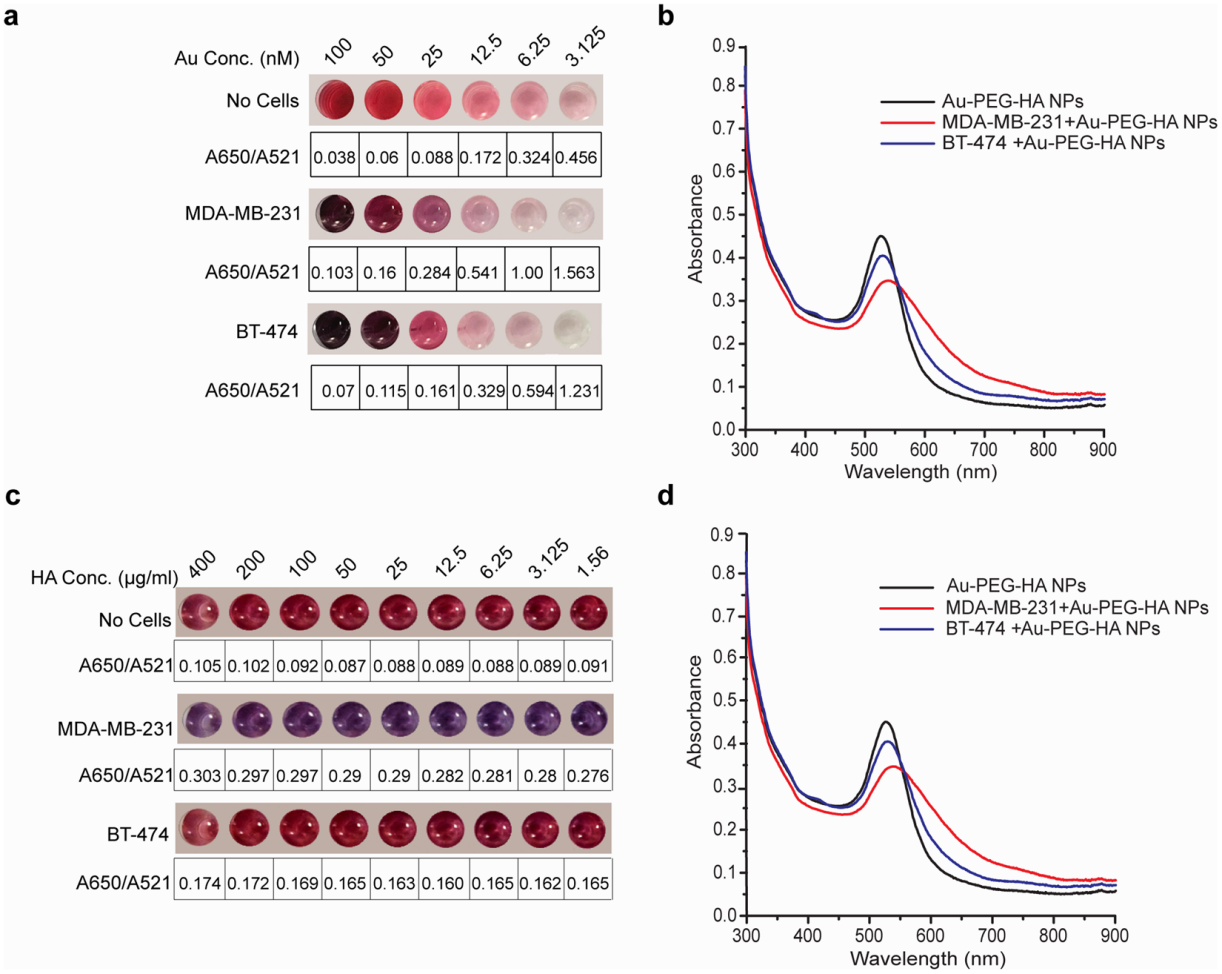
Optimization of Au and HA concentration in Au-PEG-HA NPs for cell detection. (**a**) Colorimetric changes (with A650/A521 ratio) of Au-PEG-HA NPs after incubation with MDA-MB-231 (50,000 cells) and BT-474 (50,000 cells) at varied Au concentration (100–3.125 nM) show pink-violet shift (521 nm–559 nm) in MDA-MB-231 and no shift in BT-474. (**b**) UV/Vis absorbance spectrum analysis of Au-PEG-HA NPs after incubation with MDA-MB-231 (50,000 cells) and BT-474 (50,000 cells) at 25 nM Au concentration. (**c**) Colorimetric changes (with A650/A521 ratio) of Au-PEG-HA NPs after incubation with MDA-MB-231 (50,000 cells) and BT-474 (50,000 cells) at varied HA concentration (400–1.56 µg/ml) and 25 nM Au. (**d**) UV/Vis absorbance spectrum analysis of Au-PEG-HA NPs after incubation with MDA-MB-231 (50,000 cells) and BT-474 (50,000 cells) at 100 HA µg/ml concentration (25 nM Au).

We first varied the concentration of Au (100–3.125 nM) and found that when 50,000 MDA-MB-231 (CD44^+ve/high^) cells are added to the NPs solution, the color of the solution changes from pink to violet (Fig. 2a), and this is accompanied by a shift of the SPR peak from 521 to 559 nm (Fig. 2b). There was a simultaneous increase of absorbance at 650 nm as well as a corresponding increase in A650/A521 ratio, which confirmed the aggregation of NPs due to the interaction of Au-PEG-HA NPs with MDA-MB-231 cells (Fig. 2b). We also observed that the colorimetric change from pink to violet was extremely saturated at the highest Au concentration tested (100 nM and 50 nM), and the intensity of the color change showed a gradual reduction with lower Au concentrations (Fig. 2a).

Next, we added the same number (50,000 in number) of BT-474 cells to the NPs solution with varied concentration of Au (100–3.125 nM) and we observed a slight decrease in the absorbance peak value (521 nm) and a minor increase in the absorbance at 650 nm indicating some level of aggregation due to nonspecific NPs-cell interaction at higher Au concentration (100 nM and 50 nM) which is also inferred from the increase in A650/A521 ratio. Here too, we observed an extremely saturated violet color at the highest Au concentration (100 nM and 50 nM), which gradually reduced with decreasing Au concentrations (Fig. 2a). Since our goal was to develop a platform to detect the specific binding to cells of interest as a function of colorimetric changes, visualization of the color change becomes a key parameter for evaluating the efficacy of this platform. Therefore, we chose the optimized Au concentration of 25 nM as this allowed for a clear observation of colorimetric changes with narrow and defined UV-Vis absorbance spectrum (with violet shift). Moving forward, the higher concentrations of Au (100 nM and 50 nM) were not considered so as to avoid color saturation as well broad absorbance spectrum (Fig. 2a). Similarly, the lower concentrations of Au (12.5 nM) was excluded due to the broad absorbance spectrum (Fig. S3a,b).

Next, we varied the concentration of HA (400–1.56 µg/ml) and kept a constant concentration of Au (optimized at 25 nM). Our data showed a change in the color of the NPs solution from pink to violet upon adding MDA-MB-231 cells (Fig. 2c) at all concentrations of HA tested. We also observed a decrease in the Au absorbance peak value when MDA-MB-231 cells (50,000 in number) were added, and this was accompanied by a shift of the SPR peak from 521 to 559 nm (Fig. 2d). Importantly, we did not observe colorimetric changes as well as A650/A521 ratio variations (Fig. 2c) for BT-474 cells. For all the subsequent experiments, the optimized HA concentration was selected to be 100 µg/ml based on the clear observation in colorimetric changes with narrow UV-Vis absorbance spectrum (with violet shift). The higher concentrations of HA (400, 200 µg/ml) were not considered due to the slightly viscous nature of HA at higher concentrations, which causes improper optical absorbance (Fig. 2c and Fig. S3c,d). To sum up, Au-PEG-HA NPs on interacting with MDA-MB-231 cells showed specific colorimetric change with a narrow UV-Vis absorbance spectrum (accompanying SPR shift from 521 to 559 nm) at optimized Au (25 nM) and HA (100 µg/ml) concentrations and such colorimetric changes were not observed with BT-474 cells.

### Au-PEG-HA NPs bind selectively to cells expressing CD44

**Figure 3:**
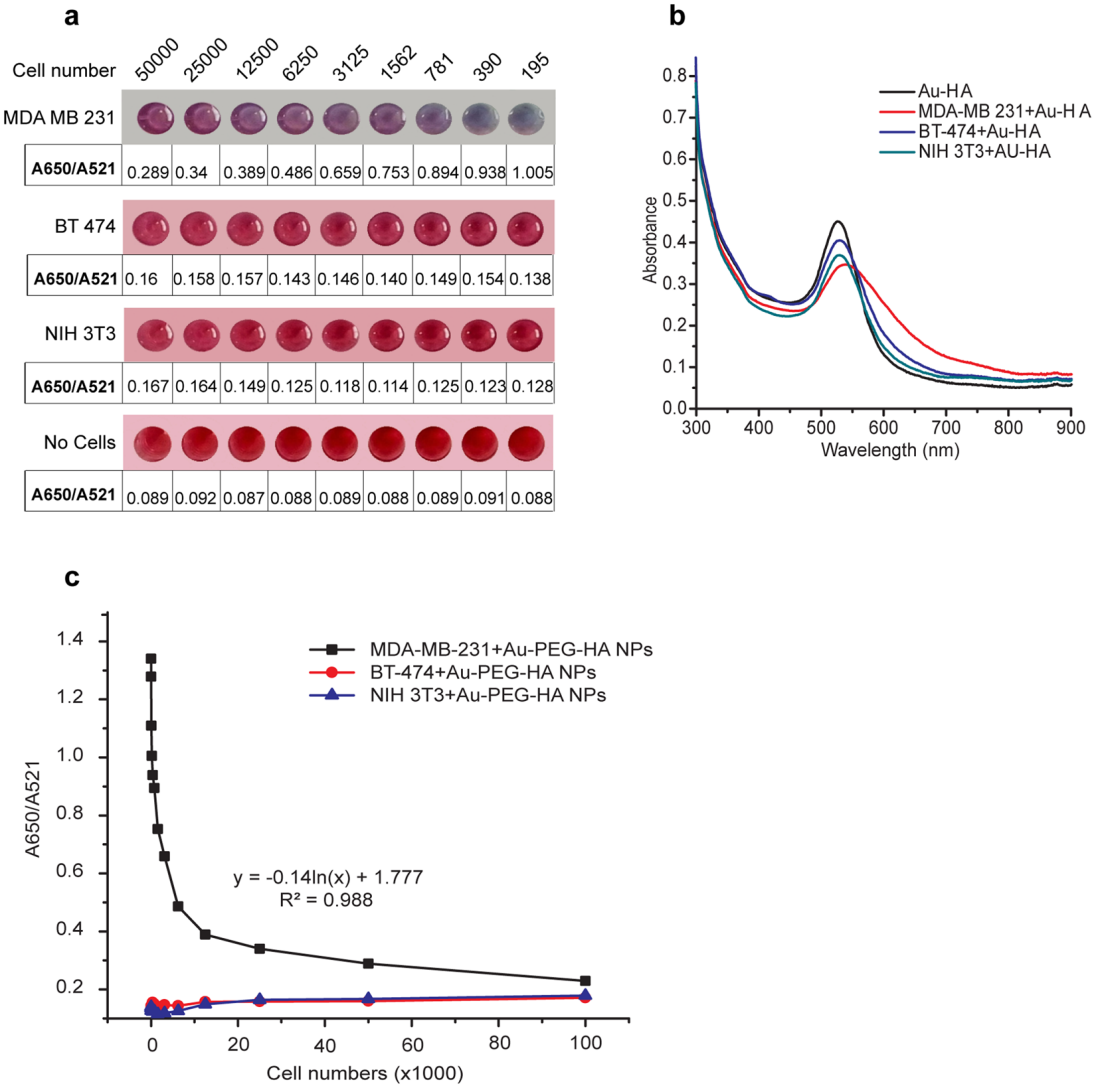
Differential identification of cells using Au-PEG-HA NPs (colorimetric changes and UV/Vis absorbance spectrum analysis). (**a**) Comparison of colorimetric changes (with A650/A521 ratio) of Au-PEG-HA NPs after interaction with different cell populations (MDA-MB-231, BT-474, NIH 3T3) at varied cell numbers (50,000–195). (**b**) UV/Vis absorbance spectrum analysis of Au-PEG-HA NPs after interaction with different cell population (MDA-MB-231, BT-474, NIH 3T3) at 50,000 cell number. (**c**) Relationship between A650/A521 ratio and cell number after incubating Au-PEG-HA NPs with MDA-MB-231, BT-474, NIH 3T3 cells for cell numbers ranging from 195 to 100000.

In order to quantify the number of cells bound to our NPs, we added varying numbers (50,000–195) of MDA-MB-231 cells (CD44^+ve/high^) to Au-PEG-HA NPs and, as shown in Fig. 3a, we observed a gradual reduction of violet color with decreasing cell numbers, indicating an inverse relationship between A650/A521 ratio and cell number. We also observed a shift of the SPR peak from 521 to 559 nm, along with a simultaneous increase in absorbance at 650 nm (higher A650/A521 ratio), which confirmed the aggregation of NPs due to a competition between the cells and the salts present in the PBS that interact with Au-PEG-HA NPs (Fig. 3b). In the case of BT-474 cells (CD44^−ve/low^) and NIH 3T3 cells (CD44^−ve^), we observed a decrease in the absorbance peak value (521 nm) and a slight increase in A650, indicating some level of nonspecific binding, but we did not see any colorimetric change (violet shift) as well as any significant variation in the A650/A521 ratio (Fig. 3a).

In order to predict the number of cells bound to the NPs as a function of the observed A650/A521 ratio, we plotted a standard curve between cell numbers and the accompanying A650/A521 ratio. For MDA-MB-231 cells, we found that a logarithmic relationship exists between the cell number and the A650/A521 ratio (R^2^ value = 0.988), confirming that CD44-HA binding governs the interaction between NPs and cells (Fig. 3c). Such relationships were not observed for BT-474 cells and NIH 3T3 cells. This helps with a quantitative estimation of cells based on an easily measured parameter, another feature relevant to any diagnostic platform.

### Exploiting CD44-HA interaction to detect cells of interest from a heterogeneous population

**Figure 4:**
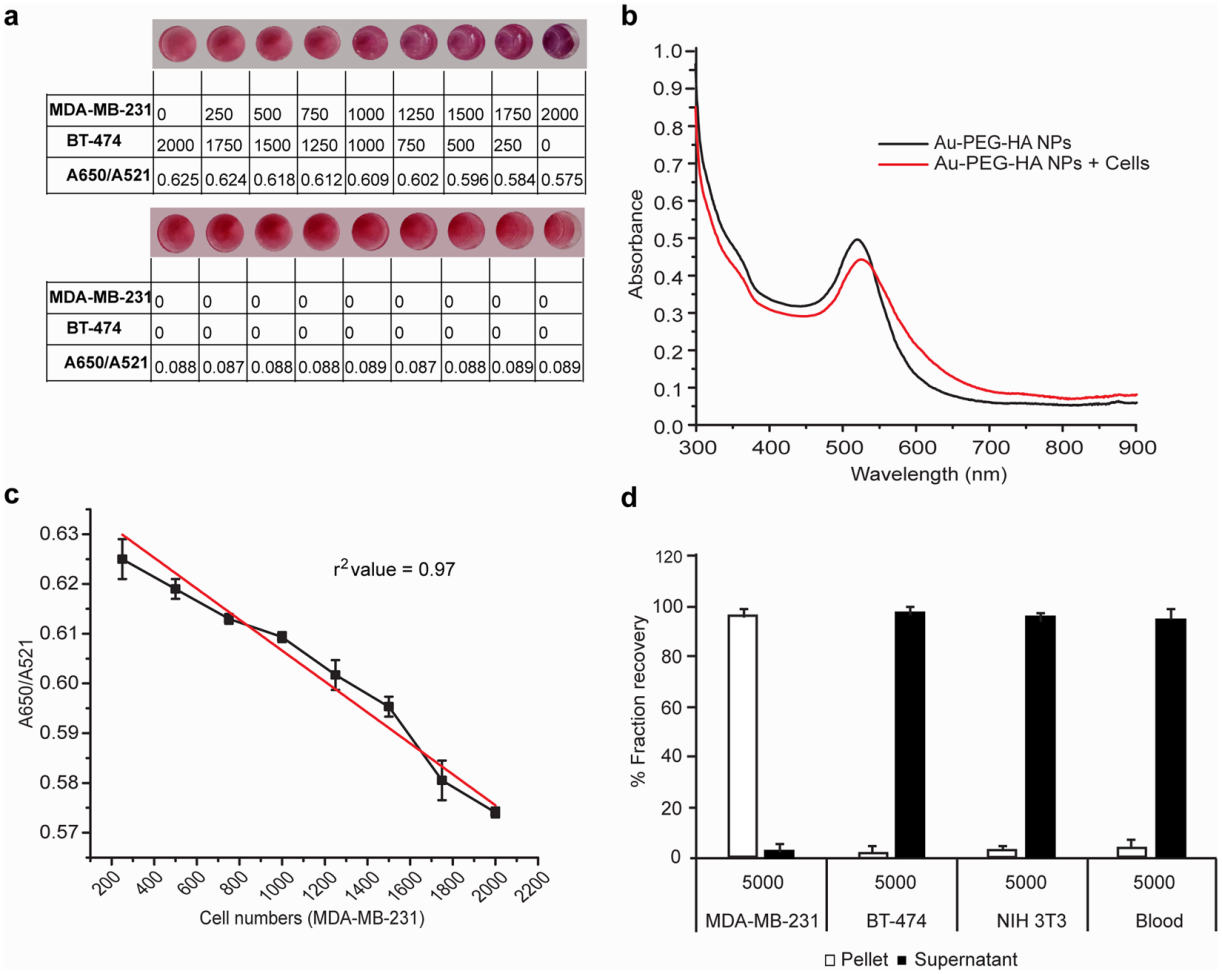
Exploiting CD44-HA interaction for detecting cells of interest (MDA-MB-231) from a heterogeneous population. (**a,b**) Cell mixtures were prepared by mixing MDA-MB-231 (CD44^+ve/high^) and BT-474 cells (CD44^−ve/low^) in varying ratios, but keeping the total number of cells constant (2000). 100 µl of Au-PEG-HA NPs solution (25 nM Au and 100 µg/ml HA) was added to each well, followed by 10 min incubation at room temperature. The colorimetric changes (with A650/A521 ratio) (**a**) and UV-Vis absorption spectra (**b**) were recorded and compared with control (Au-PEG-HA NPs) values. (**c**) Variation in A650/A521 ratio of Au-PEG-HA NPs after incubation with cell mixture with respect to target cell population (MDA-MB-231). (**d**) Flow cytometric analysis of cells isolated from AU-PEG-HA NPs using differential centrifugation.

The underlying principle of this platform is the exploitation of the ligand-receptor binding chemistry for cell detection/isolation. Specifically, we postulate that in a mixed-cell population, the NPs will selectively bind to cells expressing CD44. In order to evaluate the efficacy of the Au-PEG-HA NPs platform for specific cell detection, cell suspensions were prepared by mixing MDA-MB-231 (CD44^+ve/high^) and BT-474 cells (CD44^−ve/low^) in varying ratios, but keeping the total number of cells constant (2000). 100 µl of Au-PEG-HA solution (25 nM Au and 100 µg/ml HA) was added to each well, followed by incubation for 10 minutes at room temperature. We observed that in the cell mixture when MDA-MB-231 cells were increased from 0 to 2000 (simultaneous decrease in BT-474 cells from 2000 to 0), we observed a clear colorimetric change (pink to violet) accompanied by a SPR shift (521 nm to 559 nm) (Fig. 4a,b). Also, the presence of MDA-MB-231 cells in the cell mixture, contributes to a slight increase in absorbance at 650 nm (as reflected from decreased A650/A521 ratio). We also observed a linear relationship between MDA-MB-231 cells added and A650/A521 ratio (R^2^ value = 0.97) (Fig. 4c). We conclude that the specificity with which Au-PEG-HA NPs bind to MDA-MB-231 cells is driven by a strong affinity of HA towards CD44, resulting in specific colorimetric changes as well as a SPR peak shift.

In a separate experiment, we added 5000 MDA-MB-231-GFP cells to Au-PEG-HA NPs and allowed the cells to interact with the NPs for 10 minutes at room temperature. After this period, the NPs-bound cells were separated as a pellet fraction by differential centrifugation (600 rpm, 30 minutes). We reasoned that if our hypothesis was correct, the vast majority of the MDA-MB-231-GFP cells would be contained in the NPs-containing pellet fraction and not in the supernatant. Additionally, if cells that have low to negative expression of CD44 such as BT-474, NIH 3T3 and blood cells are added to the NP solution, we expected the vast majority of these cells to be present in the supernatant and not in the pellet fraction. FACS analysis of the cells present in both the pellet and the supernatant revealed that 96.87 ± 2.80% MDA-MB-231-GFP cells were found in pellet fraction. Interestingly, in the case of BT-474/NIH3T3/Blood, the percentage of viable cells in the supernatant was found to be 98.33 ± 1.67, 96.67 ± 3.33 and 95.77 ± 4.23 respectively (Fig. 4d). Therefore, we concluded that Au-PEG-HA NPs selectively and specifically bind to MDA-MB-231 cells via CD44-HA interaction. HA can act as a targeting carrier coating on NPs because it is a water soluble, non-immunogenic polysaccharide with specific functional groups available for chemical conjugation. Here, we have shown that the strong affinity binding of HA and CD44 can be used to target and isolate breast cancer cells from a mixed cell population. We recognize that CD44 is expressed by a variety of cell-types; however, only the activated form of CD44 is expressed by cancer cells^42,43^. Therefore, CD44, especially when used in conjunction with other cancer cell-specific markers, can be a valuable tool in targeted cancer therapy applications^21,24^ and diagnostic applications like CTC enumeration.

### Au-PEG-HA NPs allow for recovery of viable cells from target population with high specificity

**Figure 5:**
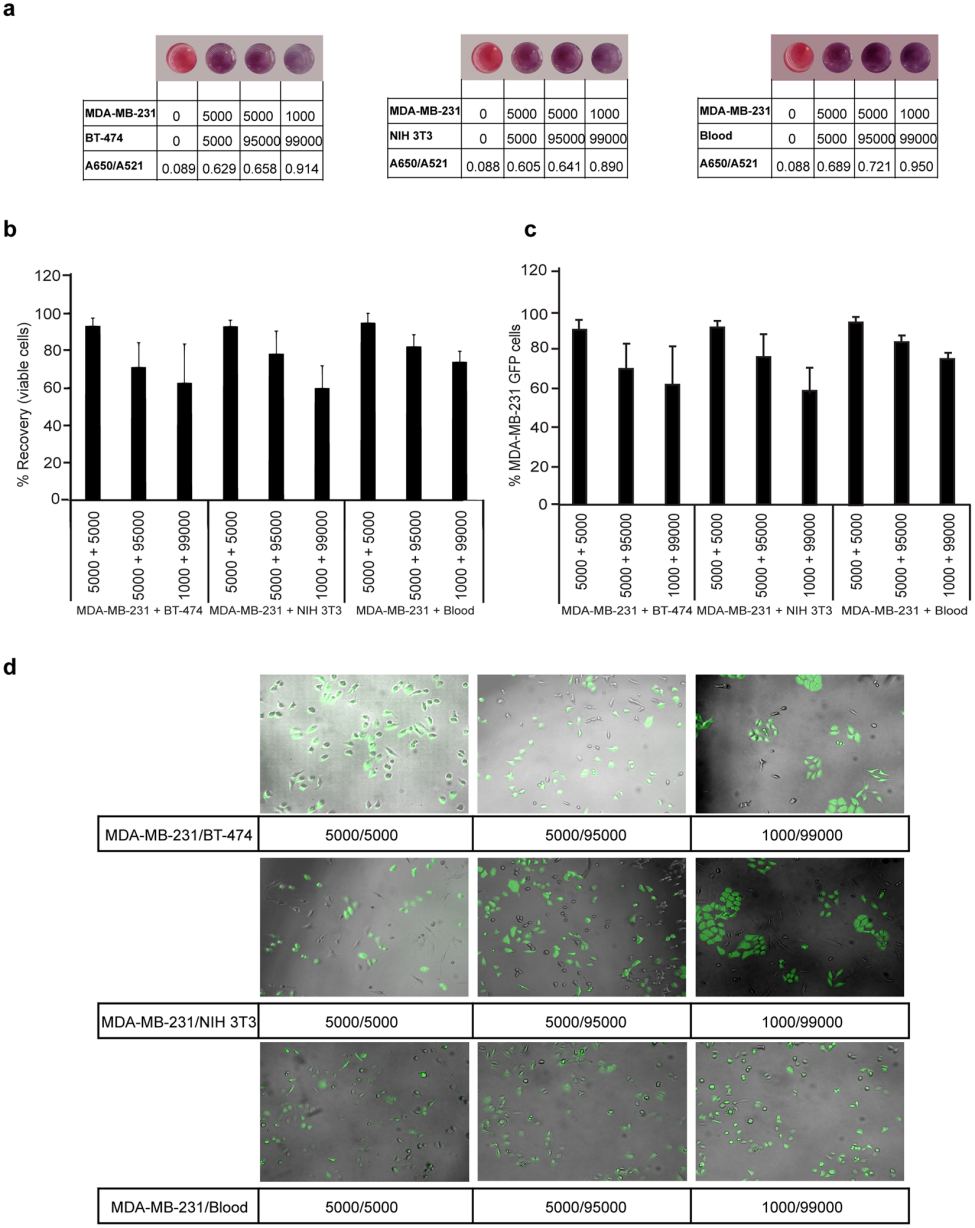
Au-PEG-HA NP-mediated recovery of viable cells of interest from a heterogeneous population. (**a**) Colorimetric changes (with A650/A521 ratio) of Au-PEG-HA NPs after interaction with a mixture of cells (MDA-MB-231 + BT-474, MDA-MB-231 + NIH 3T3, MDA-MB-231 + Blood cells) with different MDA-MB-231 cell densities (50%, 5%, 1% of MDA-MB-231 cells). (**b**) Recovery of viable MDA-MB-231 cells from a mixture of spiked MDA-MB-231 cells with BT-474/NIH 3T3/Blood cells at different cell densities (50%, 5%, 1% of MDA-MB-231 cells). The cell mixtures were treated with Au-PEG-HA NPs for 10 min, and then separated by differential centrifugation (600 rpm/30 min). The cells were disassociated from NPs by vortex and checked for viability by the trypan blue live/dead cell counting assay. (**c**) Recovery of viable MDA-MB-231 cells from a mixture of spiked MDA-MB-231-GFP cells with BT-474/NIH 3T3/Blood cells at different cell densities (50%, 5%, 1% of MDA-MB-231 cells) followed by a 10 minute incubation with Au-PEG-HA NPs and separation by differential centrifugation (600 rpm/30 min). The isolated cells were evaluated by FACS analysis. (**d**) Representative merged brightfield and fluorescent microscopy images of MDA-MB-231-GFP cells cultured from recovered viable cells after 24 hr incubation.

In routine laboratory experiments, the relevance of this platform can be measured by two aspects: (a) how effectively can it ‘sort’ the cells of interest from a heterogeneous population? and, (b) are the ‘sorted’ cells viable for further downstream processing? To answer these questions we mixed MDA-MB-231 cells with BT-474/NIH 3T3/Blood at varying densities (50%, 5%, and 1% of MDA-MB-231 cells). The intention of including blood cells here was to increase the application scenario of our NPs platform (assessing whether it can be employed for detecting specific cells, like circulating tumor cells, present in the blood). In each scenario, the absorbance peak value decreased and was accompanied by a violet shift of the SPR peak from 521 to 559 nm and a simultaneous increase in absorbance at 650 nm, which confirmed the aggregation of NPs due to the interaction between NPs and MDA-MB-231 cells and an increase in the A650/A521 ratio (Fig. 5a and Fig. S4a–c). The isolation and recovery of target cells was evaluated in a mixed population of cells, where MDA-MB-231 cells were spiked into BT-474/NIH 3T3/Blood cells at different percentage (50%, 5% and 1% of MDA-MB-231 cells). The cell mixtures were then incubated with Au-PEG-HA NPs (25 nM Au, 100 µg/ml HA) for 10 minutes at room temperature. Subsequently, the cells bound to the NPs were separated by differential centrifugation (600 rpm/30 min). The resulting pellet, which contains NPs-bound cells, was disassociated by gentle vortexing and the cells were checked for viability by the trypan blue live/dead cell counting assay. We observed 74–88% recovery of viable cells from the mixed populations (Fig. 5b). In the mixture of MDA-MB-231 and BT-474 cells, the cell viability was found to be 83.24 ± 0.45%, 74 ± 1.73% and 79.70 ± 3.15% at 50%, 5% and 1% of MDA-MB-231 cells in the mixed cell populations respectively (Fig. 5b). Similarly, in the MDA-MB-231 and NIH 3T3 mixture, the cell viability was 88.50 ± 1.78%, 88.46 ± 1.19% and 78.63 ± 10.96% at 50%, 5% and 1% of MDA-MB-231 cells in the mixed cell populations respectively. Finally, in the mixture of MDA-MB-231 and blood cells, the cell viability was found to be 84.85 ± 1.62%, 79.39 ± 2.23% and 78.52 ± 3.06% at 50%, 5% and 1% of MDA-MB-231 cells in the mixed cell populations respectively.

It can be argued that the trypan blue live/dead cell count assay provides information about total cell viability and this data likely includes viability of the negative cell population captured due to nonspecific interactions with the NPs. So, to specifically evaluate the viability of the cells of interest, MDA-MB-231-GFP cells were spiked in to BT-474/NIH 3T3/Blood cell suspension at varying concentrations (50%, 5% and 1%). These cells were then incubated with the Au-PEG-HA NPs for 10 minutes at room temperature and subsequently subjected to differential centrifugation (600 rpm/30 min). The recovered cell pellet was then disassociated via gentle vortexing, and the viability of MDA-MB-231-GFP was evaluated by counting GFP-positive cells through FACS analysis.

FACS data (Fig. 5c) showed that when MDA-MB-231-GFP constituted 50% of the cells in the cell mixture (5000:5000), the viability of the cells recovered from Au-PEG-HA NPs was 92.73 ± 4.43% (with BT-474), 92.54 ± 3.51% (with NIH 3T3) and 94.45 ± 5.25% (with blood cells). When the MDA-MB-231-GFP cells were reduced to 5% in the cell mixture (5000:95000), the percentage of GFP-positive cells was 70.87 ± 13.10% (with BT-474), 78.03 ± 12.24% (with NIH 3T3) and 81.90 ± 6.49% (with blood cells). On further reduction of MDA-MB-231-GFP cells to 1% in the cell mixture (1000:99000), the percentage of GFP-positive cells was decreased to 62.39 ± 21.03% (with BT-474), 59.74 ± 11.95% (with NIH 3T3) and 73.60 ± 5.79% (with blood cells). We concluded that upon spiking MDA-MB-231 cells (high CD44 expression) with BT-474/NIH 3T3/blood cells (low/negative expression of CD44) at different cell density (50–1%), we are able to isolate MDA-MB-231 cells with 60–94% efficiency .

The relatively wide range of recovery of the cells of interest is driven by nonspecific binding of BT-474, NIH 3T3 and blood cells with Au-PEG-HA NPs. TEM analysis revealed the size range of the Au-PEG-HA NPs to be around 67.98 ± 20.4 nm, indicating their polydispersed nature. This alludes to the presence of Au NPs and Au-PEG NPs in the Au-PEG-HA NPs group. In future work, NPs not conjugated with HA can be eliminated by gradient centrifugation^44^, giving us a more uniform size distribution of the Au-PEG-HA NPs, leading to a reduction in nonspecific binding. In this study we have used high molecular weight HA. It has been suggested that the use of low molecular weight HA would increase the specificity of the CD44-HA interaction due to increase in cell adhesion through their associations with CD44 clustering and cytoskeletal proteins^45^. More focus on surface engineering can also reduce the nonspecific interaction by blocking free PEG on the surface of NPs with a polyanionic polymer, as cells being negatively charged will have a lesser tendency to bind this polyanion^46^.

Finally, in order to assess whether the cells recovered from the NP platform retain their morphology and phenotype in culture, we cultured them for up to 24 hours in DMEM + 10% FBS. Our data showed that MDA-MB-231-GFP cells retain their morphology in culture, leading us to conclude that they can be used in further downstream assays (Fig. 5d).

Viability of the isolated cells for further processing is a key attribute for any cell-sorting platform. Our data showed the viability of the isolated cells of interest (MDA-MB-231) from a heterogeneous cell population (BT-474/NIH 3T3/blood) to be between 60–94% depending upon various experimental conditions (spiking density of 50%, 5% and 1% of MDA-MB-231 cells). This compares favorably to the reports in the literature. For example, Au NPs were used to capture CTCs by making two-dimension nanostructured substrates, either periodic Au nanostructure array^47^ or Au NP-deposited surface^48–51^ showing wide range of cell capture efficiency (61–92%). Several approaches have been reported in the literature for similar applications. Dong *et al*., 2017 achieved 80% recovery of spiked viable PC-3 cells in a heterogeneous population of cells by using self-floating hollow glass microspheres (HGMSs) coated with a degradable nanolayered film and conjugated with capture antibodies^32^. Among other recent studies, a miniaturized lab-on a-chip (LOC) platform developed by the integration of microfluidics, electronics, and inkjet printing, was able to isolate the MDA-MB-231 cells with 79% separation efficiency^52^. A layer-by-layer (LbL) assembly of biodegradable nano-film (biotin-modified alginate and HA) and its coating along with anti-EpCAM antibody on the surface of a microfluidic chip (herringbone CTC chip) was capable of selectively isolating cancer cells from whole blood with 80% of capture efficiency^53^. There are also various other reports where comparable viable cell capture efficiency was noticed ranging from 50 to 95% using different platforms^54–57^. Almost all of these systems require the use of antibody for cell isolation and also involve cumbersome techniques. Our NPs-based platform is as effective as the ones reported in literature for cell isolation, while utilizing a small molecule (HA), thereby further reducing the overall cost of the platform.

## Conclusion

In summary, here we have described a NPs-based platform for cell detection and isolation that works by exploiting the high affinity of ligand-receptor binding. The colorimetric change from pink to violet upon the binding of the cells of interest to the Au-PEG-HA NPs is the underlying principle of this platform. The violet/red shift of the SPR maximum is a consequence of a decrease/increase in the refractive index surrounding the Au NPs due to NPs-cell interaction. This makes it ideally suited for diagnostic devices, which rely on easily assessing the presence/absence of the desired parameter. Further, we have demonstrated that the platform is effective in isolating cells of interest even at 1% density, and it compares favorably, both in terms of specificity as well as cell viability, with current platforms described in the literature. We envision that continual improvements in small molecule screening will allow for incorporation of multiple ligand-receptor combinations on Au NPs (for example, DUPA-PSMA^58^, Folic acid-FRα^59^), thereby improving the detection limit of a platform such as the one described here beyond 0.01%, making it useful for detecting circulatory tumor cells in blood. Our platform also provides a convenient and cost-efficient alternative for cell sorting in laboratories and for isolation of rare populations of cells that is not feasible using existing technologies such as FACS or MACS. Importantly, cell viability with this platform is as high as 70–90%, which is conducive for subsequent manipulation of cells, including culturing and performing molecular diagnosis and other downstream analysis.

## Electronic supplementary material

Supplementary Information
