# Supplementary material for: Gold nanoparticle-based rapid detection and isolation of cells using ligand-receptor chemistry
Source: Sci Rep. 2018 Feb 13;8:2893. doi: 10.1038/s41598-018-21068-8 (PMC5811494; doi:10.1038/s41598-018-21068-8)
Supplement: Supplementary file 1 — Supplementary Information [file 41598_2018_21068_MOESM1_ESM.docx]

**Gold nanoparticle-based rapid detection and isolation of cells using ligand-receptor chemistry**

**Pradipta Ranjan Rauta^ϯ^, Pavan M. Hallur^ϯ^, Aditya Chaubey***

Anti-Cancer Technologies Program, Mazumdar Shaw Center for Translational Research, NH Health City, Hosur Road, Bangalore 560 099, India

^ϯ^ These authors contributed equally

*Corresponding author: aditya.chaubey@ms-mf.org


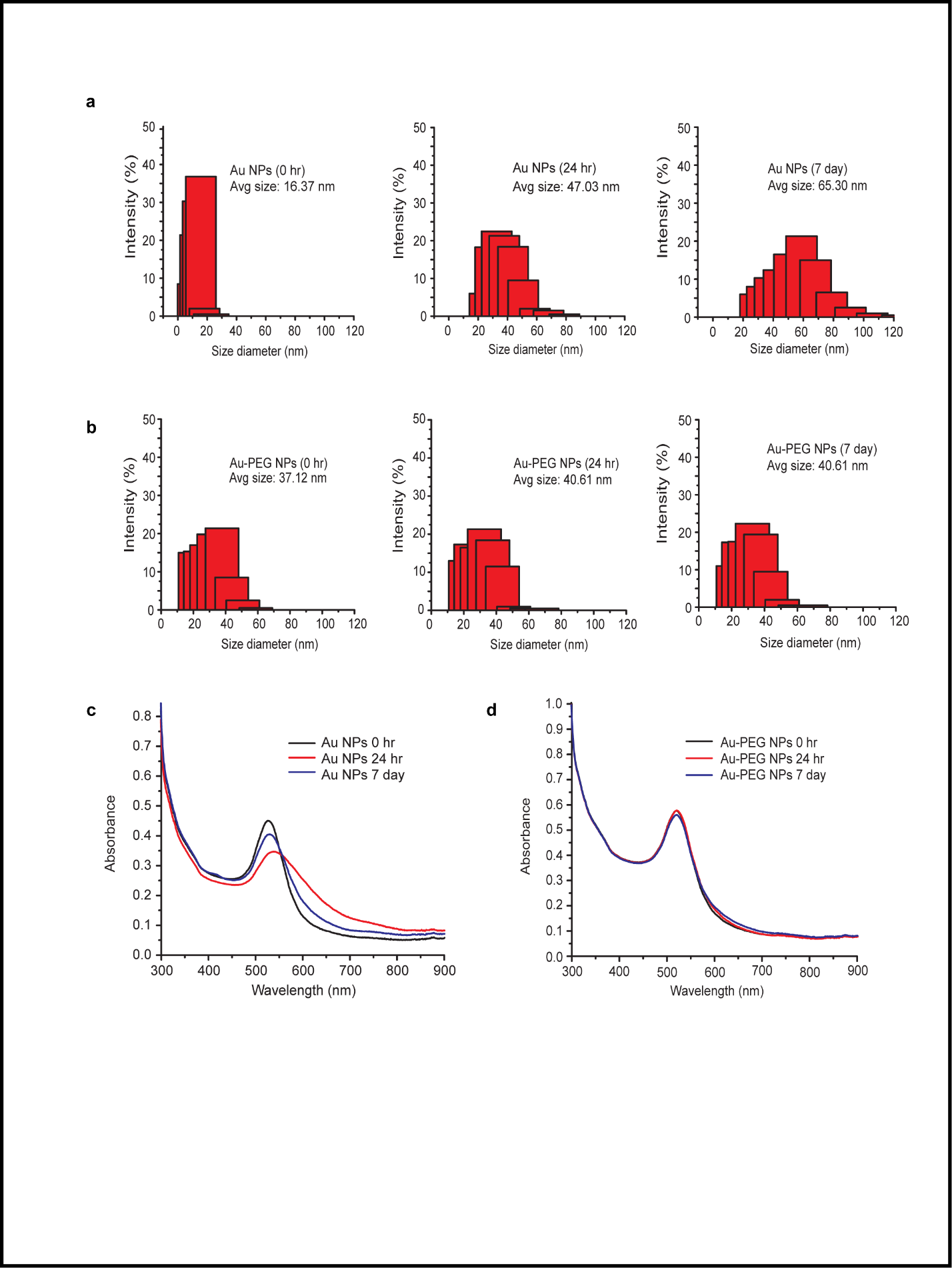
 **Supplementary Fig. S1ǀ Aggregation of Au NPs and Au-PEG NPs.** (a-b) Size distribution pattern of Au NPs and Au-PEG NPs upon incubation at room temperature at 0 hour, 24 hours and 7 days as determined from dynamic light scattering (DLS) method. (c-d) UV/Vis absorbance spectrum analysis of Au NPs and Au-PEG NPs upon incubation at room temperature for 0 hour, 24 hours and 7 days.


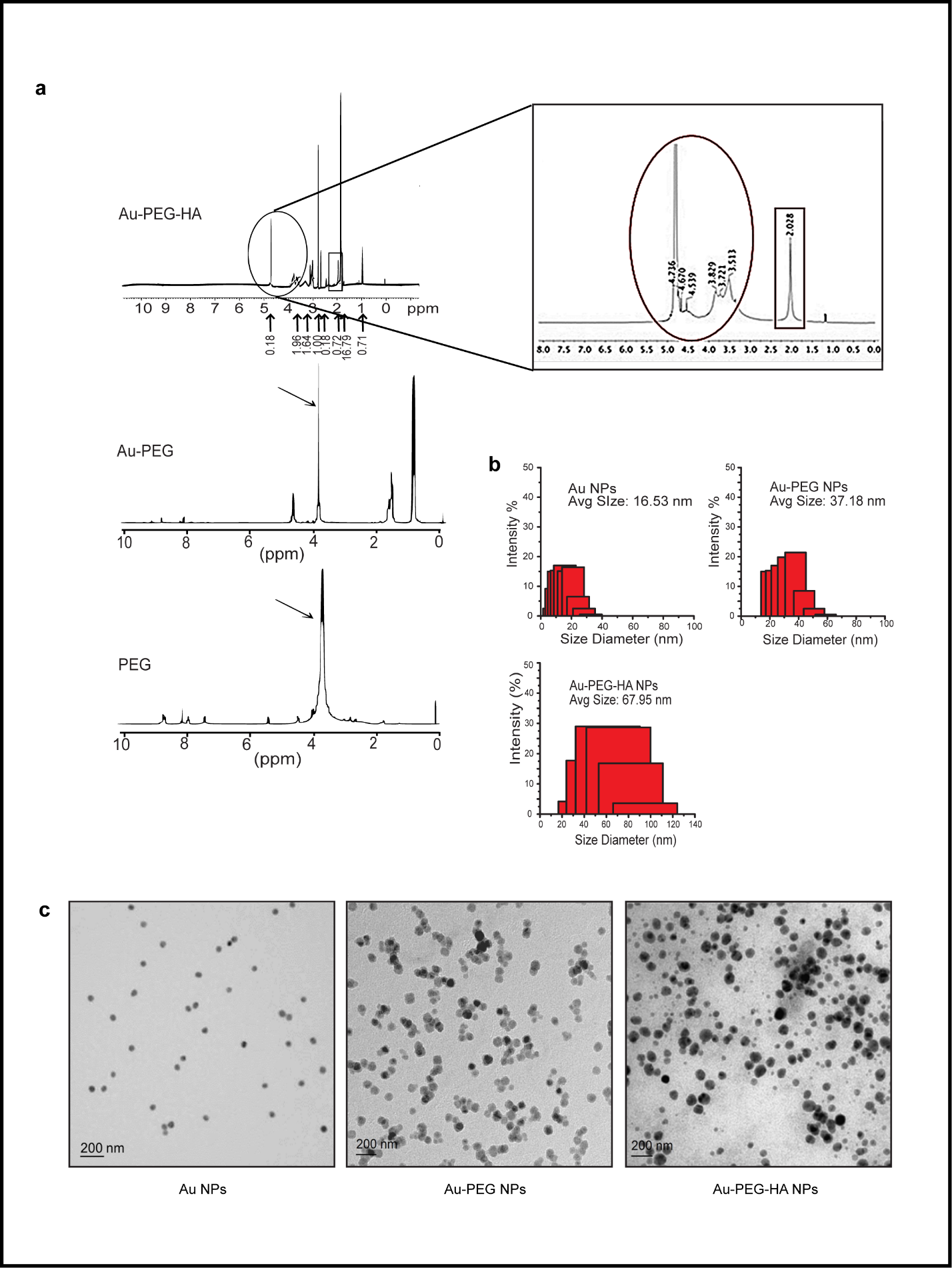
 **Supplementary Fig. S2ǀ Physico-chemical properties of Au NPs, Au-PEG NPs, Au-PEG-HA NPs.** (a) Inset of image 1b: 1H NMR spectroscopy showing characteristic peaks of HA; broad signal (3.0 -3.8 ppm): protons in the sugar rings and 4.6 ppm: two anomeric protons attached to the carbons adjacent to the two oxygen atoms (highlighted in oval); 2.02 ppm: methyl (-CH_3_) protons of the N-acetyl group of HA (highlighted in rectangular). (b) Size distribution pattern of synthesized NPs Au NPs, Au-PEG NPs, and Au-PEG-HA NPs as determined via dynamic light scattering (DLS) method. (c) Surface morphology of NPs characterized by TEM.


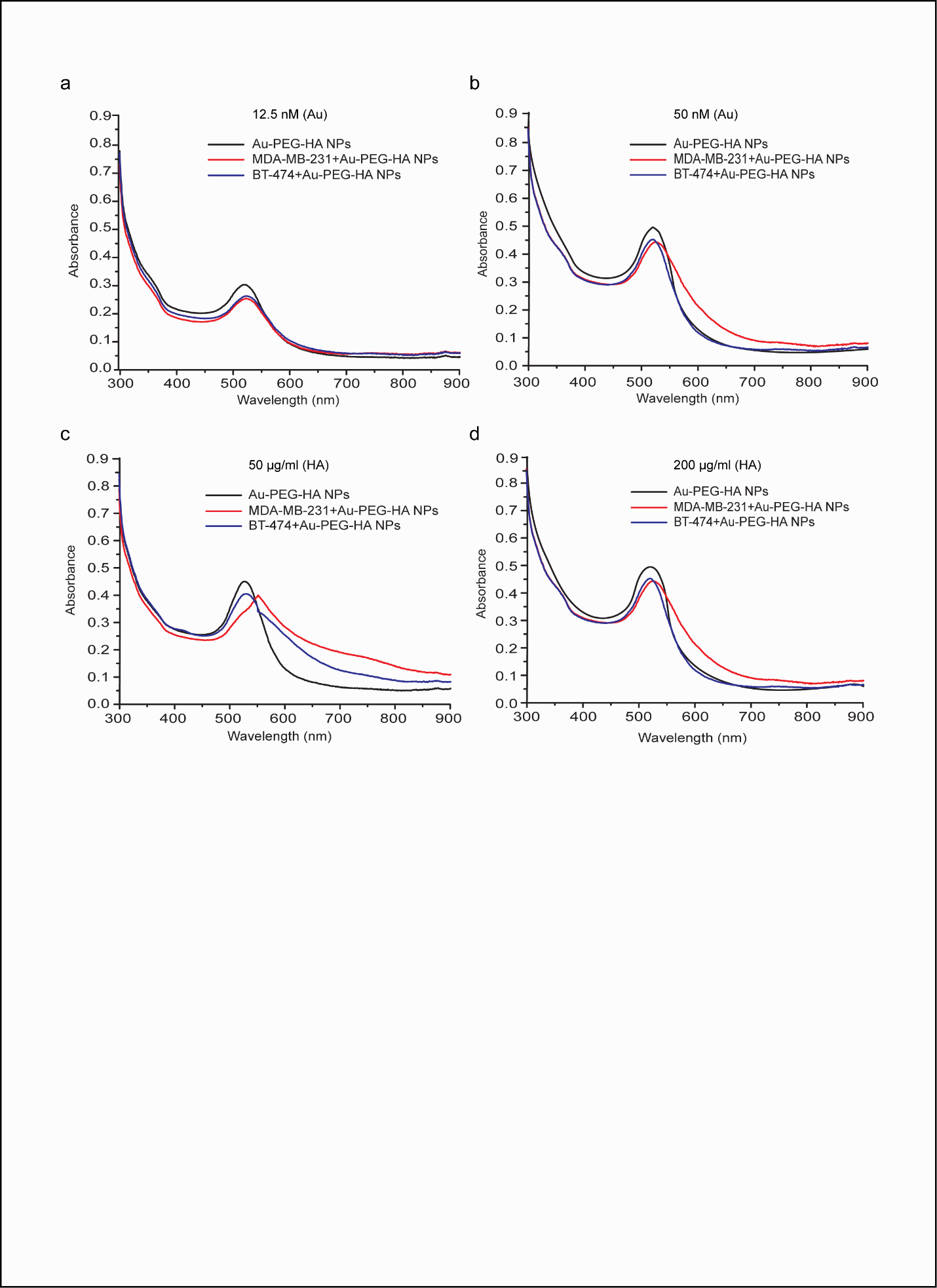
 **Supplementary Fig. S3ǀ Optimization of Au and HA concentration of Au-PEG-HA NPs.** (a-b) UV/Vis absorbance spectrum analysis of Au-PEG-HA NPs after incubation with MDA-MB-231 (50,000 cells) and BT-474 (50,000 cells) at 12.5 nM (a) and 50 (nM) (b) Au concentration. (c-d) UV/Vis absorbance spectrum analysis of Au-PEG-HA NPs after incubation with MDA-MB-231 (50,000 cells) and BT-474 (50,000 cells) at 50 µg/ml (c) and 200 µg/ml (d) HA concentration (25 nM Au).


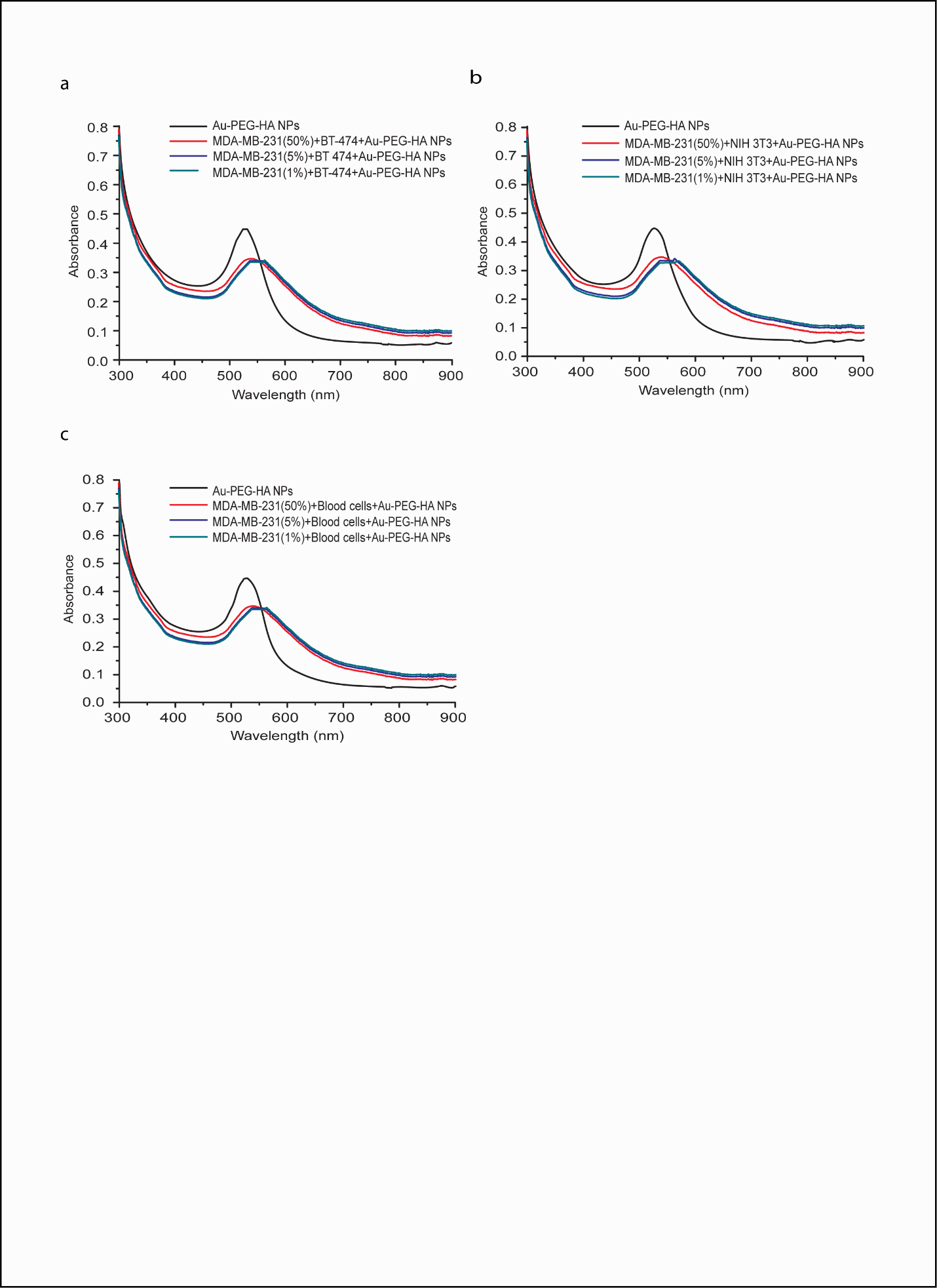
 **Supplementary Fig. S4ǀ Au-PEG-HA NPs-mediated recovery of viable cells of interest from a heterogeneous population.** (a-c) UV absorption spectra of Au-PEG-HA NPs after interaction with a heterogeneous population of cells [MDA-MB-231+BT-474 (a), MDA-MB-231+NIH 3T3 (b), MDA-MB-231+Blood cells (c)] at different cell densities (50%, 5%, 1% of MDA-MB-231).
